# Optimizing makespan and resource utilization in cloud computing environment via evolutionary scheduling approach

**DOI:** 10.1371/journal.pone.0311814

**Published:** 2024-11-22

**Authors:** Faten K. Karim, Sara Ghorashi, Salem Alkhalaf, Saadia H. A. Hamza, Anis Ben Ishak, S. Abdel-Khalek

**Affiliations:** 1 Department of Computer Sciences, College of Computer and Information Sciences, Princess Nourah Bint Abdulrahman University, Riyadh, Saudi Arabia; 2 Department of Information Technology, College of Computer, Qassim University, Buraydah, Saudi Arabia; 3 Department of Computer Science, College of Science and Humanities, Prince Sattam Bin Abdulaziz University, Al-Kharj, Saudi Arabia; 4 Department of Quantitative Methods, Higher Institute of Management, University of Tunis, Tunis, Tunisia; 5 University of Manouba, ESCT, QuAnLab LR24ES21, Campus Universitaire, Manouba, Tunisia; 6 Department of Mathematics and Statistics, College of Science, Taif University, Taif, Saudi Arabia; Effat University College of Engineering, FRANCE

## Abstract

As a new computing resources distribution platform, cloud technology greatly influenced society with the conception of on-demand resource usage through virtualization technology. Virtualization technology allows physical resource usage in a way that will enable multiple end-users to have similar hardware infrastructure. In the cloud, many challenges exist on the provider side due to the expectations of clients. Resource scheduling (RS) is the most significant nondeterministic polynomial time (NP) hard problem in the cloud, owing to its crucial impact on cloud performance. Previous research found that metaheuristics can dramatically increase CC performance if deployed as scheduling algorithms. Therefore, this study develops an evolutionary algorithm-based scheduling approach for makespan optimization and resource utilization (EASA-MORU) technique in the cloud environment. The EASA-MORU technique aims to maximize the makespan and effectively use the resources in the cloud infrastructure. In the EASA-MORU technique, the dung beetle optimization (DBO) technique is used for scheduling purposes. Moreover, the EASA-MORU technique balances the load properly and distributes the resources based on the demands of the cloud infrastructure. The performance evaluation of the EASA-MORU method is tested using a series of performance measures. A wide range of comprehensive comparison studies emphasized that the EASA-MORU technique performs better than other methods in different evaluation measures.

## 1. Introduction

Cloud computing (CC) signifies a recognized shared-computing technology that actively transports measurable services on-demand over the worldwide network [1]. CC presented consumers with endless and assorted virtual resources that can be achieved on-demand and by different billing values [2]. In addition, the CS (task scheduling (TS)) is also defined independently as a task mapping measure on a group of accessible sources in the cloud context for implementation in consumers’ definite QoS limitations (makespan and cost). Workflows (general uses are linked with experimental research such as earthquake, biology, and astronomy) were moved to the CC for implementation [3]. In CC, job scheduling, TS, or resource selection are the most extensive complications, which store cloud users’ and service providers’ attention. An exact study on TS workings also reproduced optimistic results [4]. The most significant task in the domain of CC is RS. When implementing an RS, a sufficient quality of service (QoS) must be preserved using suitable hardware structures and techniques [5]. A module of CC structure, generally denoted as an agent in modern work, is highly liable for recording the demands of end-consumers about the accessible virtualized hardware that is executed in a type of virtual machine (VM). The cloud agent executes mapping by implementing the scheduling model [6].

The number of delivered tasks and many available resources make it challenging to map challenges to the proper VM for implementation. Few VMs are under-used and over-used if the unsuitable scheduling model is employed, and the suggestion of such states is performance degradation of the CC method as a complete [7]. The RS issue goes to the set of NP hard problems. The cloudlet and TS are assumed to be used for the mapping procedure to accept the task of the end-consumer to the accessible VM. Numerous models and approaches for RS in CC atmospheres are obtainable [8]. For example, a few methods have been utilized, such as traditional (deterministic) techniques. However, conventional optimizer models could be more effective since deterministic techniques do not make satisfactory, nor-optimum, or near-optimum solutions within sufficient computation time for NP-hard tasks [9]. Based on the search space complication and the number of plausible outcomes, conventional techniques could not assess each latent solution from the search area in a polynomial time [10].

This study develops an evolutionary algorithm-based scheduling approach for the makespan optimization and resource utilization (EASA-MORU) technique in cloud infrastructure. The EASA-MORU method aims to maximize the makespan and effectually use the resources in the cloud framework. In the EASA-MORU technique, the dung beetle optimization (DBO) technique is used for scheduling purposes. Moreover, the EASA-MORU technique balances the load properly and distributes the resources based on the demands of the cloud infrastructure. A wide range of comprehensive comparison studies highlighted that the EASA-MORU technique performs better than other methods in terms of different evaluation measures.

- The method is constructed to balance the workload across cloud infrastructure by intelligently dispersing tasks among resources. This ensures that each resource is employed optimally, improving complete accomplishment and efficiency. By dynamically altering the allocation of resources based on current demands, it maintains a stable and responsive system that adapts to fluctuating workloads in real time.
- The presented technique optimizes resource dispersal within cloud infrastructure by aligning resource allocation with the requirements and demands of ongoing tasks. This proactive model enhances resource consumption and ensures critical tasks receive adequate resources.
- The technique effectively schedules tasks within cloud environments by employing the DBO model’s advanced optimization abilities. The approach’s fast convergence allows for swift decision-making, reduced latency, and optimized task allotment.
- The technique’s robustness is portrayed by its capacity to efficiently handle convolutional real-world threats in scheduling and resource allotment. Adapting to various scenarios and optimizing resource management strategies ensures reliable accomplishment across several operational conditions, thereby assisting seamless and effective cloud infrastructure operations.
- The scheduling technique presents a novel cloud infrastructure demand scheduling model by incorporating the DBO model. It improves the effectiveness of the search and global exploration abilities via non-linear improvements, thus making it more adept at resolving convolutional real-world issues.

The remaining sections of the article are arranged as follows: Section 2 offers the literature review, and section 3 represents the proposed method. Then, section 4 elaborates on the results evaluation, and section 5 completes the work.

## 2. Literature review

Alruwais et al. [11] developed a Farmland Fertility Algorithm based RS for the Makespan Optimizer (FFA-RSMSO) in CC atmosphere. The projected model was dependent upon FFA, which, by the farmland fertility naturally, where the agriculturalists divide the numerous farm regions dependent upon the superiority of soil, and thus, each area’s soil superiority is separate from others. Furthermore, based on the request, the projected FFA-RSMSO method was used for load balancing (LB) and even spreading sources. In [12], a hybrid Whale optimizer algorithm (WOA)-based MBA technique has been presented for resolving the multi-object TS issues in the CC platform. Also, in this method, the multi-object behaviour reduces the makespan by enlarging the source application. Banerjee et al. [13] present a 3-step model. First, a precedence graph is built by analyzing the relations among tasks. Next, assigning works to servers changed the priority graph into the dual machine Johnson Sequence issue. Lastly, the Dynamic Heuristic Johnson Sequence technique defines the finest order of works on every server, thus efficiently reducing the makespan. Mangalampalli et al. [14] present a new multi-object workflow scheduling technique utilizing DRL. Firstly, the priority of every workflow intended depends upon their dependencies, and the computed priority of the VM depends on the electricity price at the datacentre to map workflow onto the exact VM. Both priorities were served to the scheduler that employs the Deep Q-Network technique to vigorously plan jobs by reflecting these priorities of challenges and VM. In [15], a new RS for big data using a Hybrid 2- GlowWorm Optimizer Algorithm (H2-GWOA) method is developed. The Mean-GWOA (MGWAO) and Improved Glowworm Swarm Optimizer Algorithm (IGSOA) techniques enhance the MapReduce structure in assorted BD. The CloudSim platform has been utilized for the simulation.

Mangalampalli et al. [16] present TS using the Cat Swarm Optimizer (CSO) model. TS was completed by computing the priority of tasks and the priority of VM at the level to plan a proper map of tasks into VM. It has been executed by employing a cloudsim simulator, and the input method was produced arbitrarily from the cloudsim for the overall electricity cost; the NASA and HPC2N tasks were used with similar workload files. Qasim and Sajid [17] developed a scheduling depending on the Firefly Algorithm (FFA) technique to resolve the IoT- TS issue in CC satisfactorily. The altered FFA-based scheduler utilizes the transfer function (TF) model and the Quantization approach to plan IoT tasks on VM in a CC atmosphere to reduce the makespan of IoT tasks. Mohammadzadeh and Masdari [18] projected a hybrid multiobjective optimizer technique, which merges the Seagull Optimizer Algorithm and Grasshopper Optimizer Algorithm (HGSOA-GOA) methods. The study uses chaotic mapping for arbitrary numbers. The study was employed to study scientific workflow scheduler issues in CC atmospheres. In this model, a Pareto front solution is nominated utilizing a knee-point model, and then it is used. Bacanin et al. [19] propose an LSTM deep learning (DL) technique. A modified PSO method utilizing metaheuristic abilities is proposed. Furthermore, the variational mode decomposition (VMD) approach is employed for data processing. [20] introduces a comparative evaluation of several metaheuristic load-balancing techniques. Predić et al. [21] present a model utilizing recurrent neural networks (RNNs). The DL techniques are improved via hyperparameter tuning via a modified PSO metaheuristic model. Also, the method implements VMD for effectual series decomposition.

In [22], a dual-phase metaheuristic model (CSSA-DE) is proposed. Firstly, computing nodes are grouped into clusters utilizing a clustering model. Then, the Sparrow Search Algorithm (SSA) with Differential Evolution (DE) technique is incorporated to effectively search for appropriate task-VM combinations. Salb et al. [23] present an AI-based model utilizing bidirectional long short-term memory (BiLSTM) neural networks. A modified sine cosine algorithm (SCA) method is also given for optimization. Saravanan et al. [24] introduce the Improved Wild Horse Optimization with Levy Flight Algorithm for Task Scheduling in cloud computing (IWHOLF-TSC) model. This model improves the Wild Horse Optimization (WHO) technique with Levy flight theory to enhance local search abilities. Behera and Sobhanayak [25] propose a hybrid Grey Wolf Optimization (GWO) and Genetic Algorithm (GA) model to tackle multiobjective task scheduling in cloud computing. This hybrid method benefits from GA’s crossover and mutation operators and presents faster convergence for large scheduling problems. Mikram, Kafhali, and Saadi [26] presents the Hybrid HEFT-PSO-GA (HEPGA) technique, which integrates Particle Swarm Optimization (PSO), GA, and HEFT-based Initialization for optimizing task scheduling and reducing makespan in cloud computing by effectually allocating tasks and improving resource consumption. Hamed et al. [27] propose a novel scheduling approach for heterogeneous cloud computing systems utilizing a cooperation search algorithm, employing meta-heuristic advantages. Singh and Chaturvedi [28] introduce a fusion GA-modified PSO technique for effective task allocation in cloud-fog computing by balancing the load of dependent activities across heterogeneous resources.

Sandhu et al. [29] propose a model that concentrates on reducing Total Execution Cost (TEC), Total Execution Time (TET), Energy Consumption (EC), and Response Time (RT). This technique utilizes Tabu Search, Bayesian Classification, and Whale Optimization models. Yadav and Mishra [30] introduce an improved ordinal optimization approach that mitigates the search space for optimal scheduling, utilizing horse race conditions and iterative techniques for minimizing makespan by effectually allocating load to the most promising schedules. Xia et al. [31] present an Adaptive Evolutionary Scheduling Algorithm (AESA) model featuring three strategies: a heuristic for effectual population initialization, a dynamic variable evaluation to optimize Pareto-front convergence, and an adaptive reward system for decision variables. Paul et al. [32] propose the Improved Artificial Rabbit Optimization with Pattern Search (IARO-PS) approach, improving the original ARO for dynamically scheduling independent tasks. It incorporates LB with IARO-PS to optimize workload dispersion and mapping onto VMs, enhancing exploration and exploitation balance. Malti, Hakem, and Benmammar [33] introduce a novel hybrid optimization algorithm (HOA) for multiobjective task scheduling in heterogeneous IaaS cloud environments, integrating flower pollination behaviour with GWO strategies and integrating evolutionary crossover operators for balancing exploration and exploitation. Damera et al. [34] propose an advanced task-scheduling algorithm that refines the Snake Optimization Algorithm (SO) technique employing sine chaos mapping, a spiral search strategy, and dynamic adaptive weights to enhance the effectiveness of the makespan and energy. Table 1 summarizes the existing studies on resource utilization using the evolutionary scheduling approach.

**Table 1:** Summary of existing studies on resource utilization using evolutionary scheduling approach.

Ref	Techniques	Metrics	Findings
[11]	Farmland Fertility Algorithm, Resource Scheduling, Load Balancing	Makespan, Resource Allocation Efficiency, Load Distribution	This model crucially enhances resource allocation effectiveness and makespan optimization in cloud computing environments, presenting superior load balancing and uniform resource dispersion related to other models.
[12]	Hybrid Whale Optimization Algorithm, Multiobjective Behavior Algorithm, Random Double Adaptive Whale Optimization Algorithm	Makespan, Execution Time, Resource Utilization, Computational Cost, Completion Time	The HWOA-based MBA model outperforms other techniques in mitigating makespan, execution time, and computational cost while attaining faster convergence in large search spaces and improving resource consumption.
[13]	Johnson Sequencing Algorithm, Dynamic Heuristic Johnson Sequencing, Precedence Graph Construction	Makespan, Resource Utilization, Scalability, Job Scheduling Efficiency	The Dynamic Heuristic Johnson Sequencing technique crucially mitigates makespan and improves resource consumption across three servers, showing remarkable scalability and efficiency in complex cloud computing job scheduling
[14]	Deep Reinforcement Learning, Priority Calculation, WorkflowSim	Makespan, Energy Consumption, Scheduling Efficiency	The presented technique crucially mitigates makespan and energy utilization, outperforming present techniques, namely Heterogeneous Earliest Deadline First, Cat Swarm Optimization, and Ant Colony Optimization in workflow scheduling
[15]	Hybrid 2-GW Optimization Algorithm, Hadoop MapReduce Framework, CloudSim Platform	Latency, Makespan, Resource Utilization, Skewness, CPU Consumption	The H2-GWOA-based scheduler outperforms conventional approaches by enhancing latency, makespan, and CPU utilization while improving resource utilization and balancing task dispersion in big data processing utilizing Hadoop MapReduce
[16]	Cat Swarm Optimization Algorithm, CloudSim Simulator, HPC2N and NASA Workloads	Makespan, Migration Time, Energy Consumption, Total Power Cost	The Cat Swarm Optimization model substantially improves task scheduling
[17]	Firefly Algorithm, Harris Hawks Optimization, Differential Evolution	Makespan, Convergence Speed, Result Quality	The Firefly Algorithm-based scheduler substantially outperforms Harris Hawks Optimization and Differential Evolution in convergence speed and makespan minimization for IoT task scheduling.
[18]	Hybrid Seagull Optimization Algorithm, Chaotic Maps, Knee-Point Method, CloudSim and WorkflowSim Tools	Makespan, Cost, Energy, Throughput, Inverted Generational Distance, Coverage Ratio	The presented model outperforms SPEA2 in multi-cloud workflow scheduling, showing an improved convergence rate.
[19]	Long Short-Term Memory, Deep Learning, Variational Mode Decomposition, Modified Particle Swarm Optimization, SHapley Additive exPlanations	Mean Squared Error, Coefficient of Determination, Mean Absolute Error, Index of Agreement	The LSTM technique with attention layers and VMD crucially outperformed other models in cloud load forecasting.
[20]	Particle Swarm Optimization, Various Metaheuristic Load Balancing Algorithms	Makespan Time, Degree of Imbalance, Response Time, Data Center Processing Time, Flow Time, Resource Utilization	The proposed technique outperforms other metaheuristic algorithms
[21]	Recurrent Neural Networks, Modified Particle Swarm Optimization, Variational Mode Decomposition, SHapley Additive exPlanations	Mean Square Error, Root Mean Square Error, Index of Agreement	The proposed RNN-based forecasting approach, optimized with a modified PSO and VMD, substantially outperforms existing techniques
[22]	Dual-Phase Metaheuristic Algorithm, Clustering Approach, Task Placement Optimization	Energy Consumption, Load Balance, Resource Leakage	The presented technique outperforms existing models by efficiently reducing energy utilization, balancing load, and mitigating resource fragmentation, illustrating greater performance in task-to-VM placement efficiency
[23]	Bidirectional Long Short-Term Memory Neural Networks, Modified Sine Cosine Algorithm	Forecast Accuracy, Comparative Analysis	The proposed AI-based forecasting technique, employing BiLSTM and optimized by a modified SCA, has substantial potential for precise cloud load predictions.
[24]	Improved Wild Horse Optimization, Levy Flight Algorithm, Improved Wild Horse Optimization with Levy Flight	Makespan, Resource Utilization, Load Balancing, Energy Efficiency	The presented technique illustrated greater performance in reducing makespan, maximizing resource utilization, and improving load balancing and energy effectiveness
[25]	Grey Wolf Optimization Algorithm, Genetic Algorithm, Hybrid GWO-GA Algorithm, Cloudsim Toolkit, Analysis of Variance	Makespan, Energy Consumption, Cost, Convergence Speed	The hybrid GWO-GA model outperforms conventional GWO, GA, and PSO approaches by mitigating makespan, energy consumption, and scheduling costs
[26]	Hybrid HEFT-PSO-GA Algorithm, Scheduling Constraints Optimization	Makespan, Resource Utilization	The HEPGA method enhances resource consumption and mitigates makepan by efficiently incorporating PSO and GA with HEFT-based Initialization.
[27]	Cooperation Search Algorithm, Comparison with Existing Methods	Makespan	The proposed technqiue outperforms NGA, GA, WOA, GSA, and HHG imodels
[28]	Hybrid GA-modified PSO, Workflow Scheduling	Makespan, Energy Consumption, Cost	The Hybrid GA-modified PSO approach substantially mitigates overall execution time and cost of workflow tasks, converges to optimum outcomes more quickly, and delivers improved quality results related to present methods.
[29]	Tabu Search, Bayesian Classification, Whale Optimization	Total Execution Cost, Total Execution Time, Energy Consumption, Response Time	The presented TBW optimization approach crucially outperforms GA-PSO and Whale Optimization
[30]	Ordinal Optimization, Linear Regression	Makespan, Overhead	The proposed ordinal optimization method, integrated with linear regression, efficiently mitigates the search space and overhead, attaining optimal schedules with lesser makespan. This technique also anticipates future dynamic workloads accurately, improving the overall effectualness of cloud task scheduling.
[31]	Heuristic Population Initialization, Variable Analysis Strategy, Adaptive Reward Strategy	Makespan, Energy Consumption	The presented technique substantially enhances makespan and energy utilization in cloud platforms, outperforming five representative baseline models. The heuristic Initialization, variable analysis, and adaptive reward strategies all contribute to its greater performance, with the adaptive reward mechanism having the greatest impact.
[32]	Improved Artificial Rabbit Optimization based on Pattern Search, Load Balancing Strategy, Benchmark Function, Simulation-Based Comparison	Makespan, Resource Utilization	The proposed model outperformed present techniques by mitigating makespan and enhancing resource utilization.
[33]	Hybrid Optimization Algorithm, CloudSim Framework, Optimization Criteria, Comparison Algorithms	Time Makespan, Resource Utilization, Degree of Imbalance, Throughput	The proposed hybrid approach portrays greater performance compared to existing models. It efficiently addresses local optimality traps and premature convergence problems, illustrating its advantages in multiobjective task scheduling within heterogeneous IaaS cloud environments
[34]	Enhanced Snake Optimization Algorithm, Sine Chaos Mapping, Spiral Search Strategy, Dynamic Adaptive Weights	Makespan, Energy Efficiency, Convergence Speed	The presented technique demonstrates enhancements in cloud scheduling performance. It also shows quicker convergence to optimum outcomes.

The existing studies for task scheduling in cloud computing each have specific limitations. Some techniques improve resource allocation and make-span optimization but may need help with scalability and adaptability to dynamic or large-scale environments. For instance, while specific techniques enhance execution time and computational costs, they often need more robustness in highly variable scenarios or generalize well across several real-world scenarios. Furthermore, methodologies that outperform in specific metrics, namely energy utilization or LB, might not handle rapidly changing cloud setups efficiently. These approaches portray substantial enhancements but need additional refinement to address the complexities of large-scale, dynamic cloud environments and growing technologies. Moreover, several optimization techniques, such as FFA, WOA, GWOA, and CSO, are utilized to address makespan optimization and resource utilization in cloud infrastructure. However, a significant research gap still exists by incorporating these models into a cohesive framework that optimizes makepan while effectively employing resources in cloud environments. The presented model aims to fill this gap by introducing a novel model that potentially integrates the strengths of various optimization models, hence giving improved accomplishment and scalability related to existing techniques.

## 3. The proposed method

**Fig 1:**
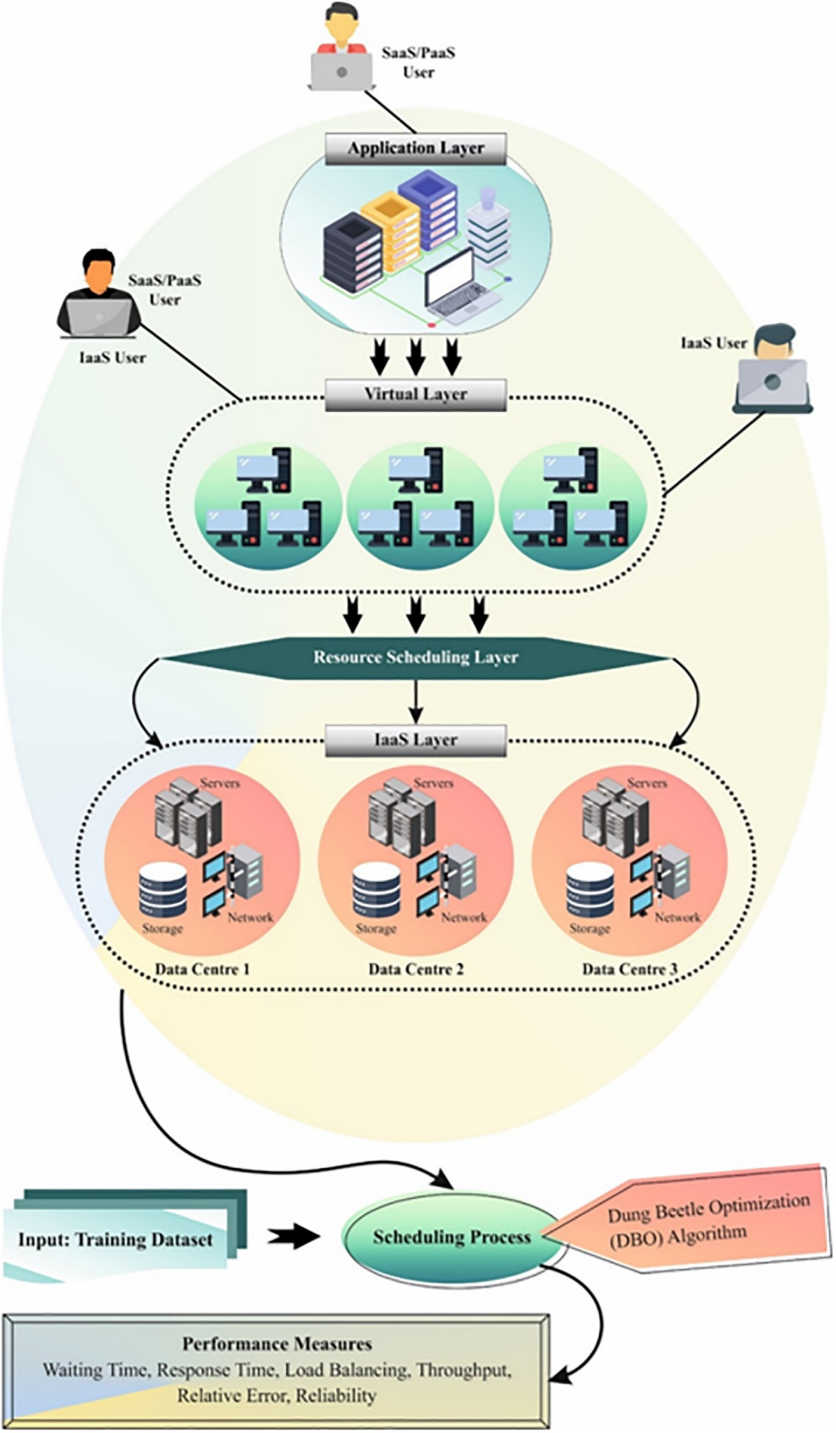
Overall flow of the EASA-MORU technique.

This work presents an EASA-MORU technique for cloud infrastructure. The EASA-MORU method aims to maximize the makespan and effectively use the resources in the cloud infrastructure. Fig 1 represents the entire flow of the EASA-MORU method.

### 3.1. System model

A cloud system can be sophisticated from one or more datacentre based on the resource requirement [35]. A sequence of datacenters (*DC*) comprises *P* datacenters to consider the public cloud system, viz., *DC* = {*dc*_1_, *dc*_2_,….*dc*_*P*_}. Each datacenter has more than one physical host (*Phs*), e.g., a datacenter *dc*_*r*_ has a series of {*ph*_*r*1_, *ph*_*r*2_,…..*ph*_*rk*_}. All the hosts have several cores to define the VM Manager (VMM), storage, memory, bandwidth, and host Million Instruction Per Second (MIPS). The VMM on the hosts are mainly liable for maintaining and managing *VMs* on the hosts.

The physical host *ph* has a series of {*vm*_1_, *vm*_2_,……..*vm*_*m*_} *vm*_*i*_ has specific configurations, for example, storage *vm*_*st*_, main memory (*vm*_*mem*_), the number of cores (*vm*_*cpus*_), and processing power (*vm*_*mips*_) in MIPS along with the cost of *vm* per hour.

The user submits their task to the service providers for processing in the cloud infrastructure without knowing the system infrastructure complexities. This task differs in requirement based on the required resources and length. The user submits *n* cloudlets or tasks {*Cl*_1_, *Cl*_2_,…….*Cl*_*n*_} for processing. The cloudlet has a certain length (*Cl*_*i*_) in Million Instruction (MI). First, the scheduler evaluates the Execution Time (*ET*) of *t*_*i*_ tasks on *each vm*_*j*_, as in the following:

$$ET\;\left(Cl_{i},\;vm_{j}\right)=\frac{\left(Cl_{i}\right)}{total_{-}MIPS\left(vm_{j}\right)}$$ (1)

In Eq (1), *total*_*mips*(*vm*_*j*_) = (*vm*_*cpus*_) * *vm*_*mips*_. Next, the scheduler evaluates the consumed power by the VMs for processing the allocated task and the performing cost in that *vm*. To reduce the task processing cost, the scheduler defines which VMs have suitable performance costs and meet the requirements of the overall tasks. Diverse *VMs* have dissimilar processing capacities since each task is processed on *a VM*, making different costs and *ET* (*Cl*_*i*_, *vm*_*j*_) for processing tasks *Cl*_*i*_ on dissimilar *VMs*. A multiobjective scheduling problem arises to maximize resource usage and reduce costs, execution time, and energy consumption.

### 3.2. Design of DBO approach

In this study, the DBO method of the EASA-MORU method is used for scheduling purposes. The behaviours of dung beetles (DBs) are motivated by foraging, dancing, rolling, breeding, and stealing [36]. At the DBO method, local searches (comprising stealing, foraging, and breeding) were employed to move the storage balls nearby to explore the optimum position. The global search describes the rolling ball behaviour, for example, rolling the feed balls to the optimum location. The working method of the proposed model modifies the DB population, evaluating the fitness function and storing the optimum individuals. Subsequently, every subpopulation has been exposed to cooperative optimization. The local search has been performed by foraging and breeding DBs. A global search could be executed with the Dancing DB and Rolling DB, and the breeding and foraging regions must be dynamically modified with the alternation in R factors to decrease the search region. Stealing DBs explore the top foraging region to increase the following. The DBO is an innovative biotic swarm intelligence (SI) optimization method developed by Jiankai in 2022. The technique has been motivated by the group activities of DB populations and considered with five various update procedures to support determining higher-quality solutions. The population classification and boundary selection approaches permit considering local convergence and global exploration. Algorithm 1 specifies the steps involved in the DBO technique.

**Algorithm 1. DBO model**.

**Input**:

 • Objective function *f*(*x*)

 • Number of dung beetles *N*

 • Maximum number of iterations *T*_*max*_

 • Search space boundaries

**Output**:

 • Optimal solution *X*_*best*_

**Steps**:

1. **Initialization**:

1.1. Randomly initialize the positions of *N* dung beetles within the search space.

1.2. Set initial positions *x*_*i*_ (0) for *i* = 1, 2, 3, ….., *N*

1.3. Evaluate the fitness of each dung beetle: *f*(*x*_*i*_).

1.4. Set *x*_*best*_ to the position of the beetle with the best fitness.

2. **For *t* = 1 to *T***_***max***_:

2.1. **Update positions**:

- For each dung beetle *i*:

- Compute the new position using:

$$x_{i}\left(t+1\right)=\;x_{i}\left(t\right)+\emptyset \times \left(x_{best}\left(t\right)-x_{i}\left(t\right)\right)+\sigma \times \left(x_{rand}\left(t\right)-x_{i}\left(t\right)\right)$$

Where *x*_*rand*_ (*t*) is in a random position in the search space.

 -∅ and *σ* are coefficients controlling exploration and exploitation

2.2. **Boundary Check**:

- Ensure *x*_*i*_ (*t* + 1) is within search space boundaries.

- If *x*_*i*_ (*t* + 1) is out of bounds, adjust it to fit within boundaries.

2.3. **Evaluate Fitness**:

- Calculate *f*(*x*_*i*_ (*t* + 1)) for each updated position.

2.4. **Update Best Solution**:

- If any dung beetle’s new fitness is better than the current best fitness:

- Update *x*_*best*_ to the position of the beetle with the best fitness.

3. **Termination**:

 • If *t* = *T*_*max*_, terminate the algorithm.

4. **Output**:

 • Return *x*_*best*_ as the optimal solution.

The DBO model follows a structured process for solving optimization issues. It begins with random Initialization, where the locations of a dung beetle populace are set within the search space, and their fitness is computed by utilizing the objective function. The technique detects the best-performing solution and upgrades the beetles’ positions iteratively, integrating the current best solution with random positions to balance exploration and exploitation. After upgrading positions, the algorithm confirms that they remain within search space boundaries. It then reexamines the fitness of these locations and upgrades the optimum solution if enhancements are found. This procedure continues until the optimum iteration number is attained. The final output is the optimum solution found, portraying the optimal or near-optimal result depending on the fitness analysis throughout the iterations.

#### 3.2.1. Ball-rolling dung beetle

The DB creates a ball of rolls and dung for the preferred position. In the rolling method, the DB must maintain the rolling dung balls directly through celestial signals (wind direction, sun location, and so on). To simulate the rolling behaviour, the DB is required to change in a specified way across the search space. The location upgrade mathematical model for rolling DBs will be given by:

$$x_{i}\left(t+1\right)=x_{i}\left(t\right)+\alpha \times k\times x_{i}\left(t-1\right)+b\times \Delta x,$$

$$\Delta x=\left|x_{i}\left(t\right)-X^{w}\right|$$ (2)

Here, *x*_*i*_(t) refers to the location data of the *i*^th^ DB at *t* iterations, *t* means the existing number of iterations; *b* denotes a constant between (0,1). The constant value *k* ∈ (0,0.2] demonstrates the coefficient of defection, and α describes a real coefficient given at −1 or 1. Therefore, −1 signifies a deviation from the new direction, and 1 means no deviation. Δ*x* has been applied to simulate the light intensity difference, and *X*^*W*^ refers to the global worst place.

While a DB addresses a difficulty from forwarding movements, it modifies the way of movement by dancing. The tangent function has been applied to mimic the dancing behaviour of DB. The dancing DB position will be upgraded as given by:

$$x_{i}\left(t+1\right)=x_{i}\left(t\right)+\text{tan}\left(\theta \right)\left|x_{i}\left(t\right)-x_{i}\left(t-1\right)\right|$$ (3)

Now *θ* ∈ [0, *π*]. When *θ* is equivalent to 0 or *π*/2, then the place of the DB is not upgraded.

#### 3.2.2. Breeding dung beetles

Particular dung balls must be concealed in somewhat protective surroundings as an egg-existing location. Hence, a boundary selection approach has been utilized for the region where female DBs place their eggs:

$$\begin{array}{l} Lb*=\text{max}\left(X^{*}\times \left(1-R\right),Lb\right),\\ Ub*=\text{min}\left(X^{*}\times \left(1+R\right),Ub\right) \end{array}$$ (4)

Here, X* characterizes the existing local optimum location, Ub* means the upper bound, Lb* represents the lower bound of the breeding field, *R* = 1 − *t*/*Tmax*, *Tmax* means the maximal count of iterations, and lower and upper boundaries of the optimizer complexity have been characterized by *Lb* and *Ub*, correspondingly.

Female DBs place their eggs in the breeding region, and every female DB can produce only one egg in every round. The boundary of the breeding region was found through the dynamically modified R-value. The location of the hatching ball at the iteration method and a mathematical model will be given below:

$$B_{i}\left(t+1\right)=X^{*}+b_{1}\times \left(B_{i}\left(t\right)-Lb^{*}\right)+b_{2}\left(B_{i}\left(t\right)-Ub^{*}\right)$$ (5)

Now *B*_*i*_(t) refers to the location data of the *i*^th^ marking ball with *t*^th^ iteration. *b*_1_ and *b*_2_ mean two independent 1 × *D* random vectors, and *D* represents the size of the optimizer problems. The hatching ball’s position is severely restricted to the breeding area. In the boundary selection approach, the brown circle denotes the existing local optimum place X*, and the blue point represents the rolling DB. Red circles have signified the upper and lower boundaries. Tiny black points denote hatching balls, which comprise only one egg.

#### 3.2.3. Small dung beetles (foraging DBs)

Eggs hatch effectively and become DBs baby. DBs have been directed for foraging by developing optimum foraging regions. The boundaries of the optimum foraging regions could be outlined as given below:

$$\begin{array}{l} Lb^{b}=\text{max}\left(X^{b}\times \left(1-R\right),\;Lb\right)\\ Ub^{b}=\text{min}\left(X^{b}\times \left(1+R\right),\;Ub\right) \end{array}$$ (6)

Here, *X*^*b*^ indicates the global optimum location. The lower and upper boundaries of the optimum searching field should be denoted as *Lb*^*b*^ and *Ub*^*b*^, correspondingly. The location of the smaller DB will be upgraded by mathematical form:

$$x_{i}\left(t+1\right)=x_{i}\left(t\right)+C_{1}\left(x_{i}\left(t\right)-Lb^{b}\right)+C_{2}\left(x_{i}\left(t\right)-Ub^{b}\right)$$ (7)

Whereas *x*_*i*_(t) represents the location data with *i*^th^ tiny DB on *t*^th^ iteration, *C*_1_ signifies an arbitrary number accompanying a standard distribution, and *C*_2_ indicates the random vector at (0,1).

#### 3.2.4. Stealing dung beetles

Particular DBs steal dung balls from alternative DBs; this method is represented as stealing DBs. In the consistent iterations, the location of the robber could be upgraded as given by:

$$x_{i}\left(t+1\right)=X^{b}+S\times g\times \left(\left|x_{i}\left(t\right)-X^{*}\right|+\left|x_{i}\left(t\right)-X^{b}\right|\right)$$ (8)

**Fig 2:**
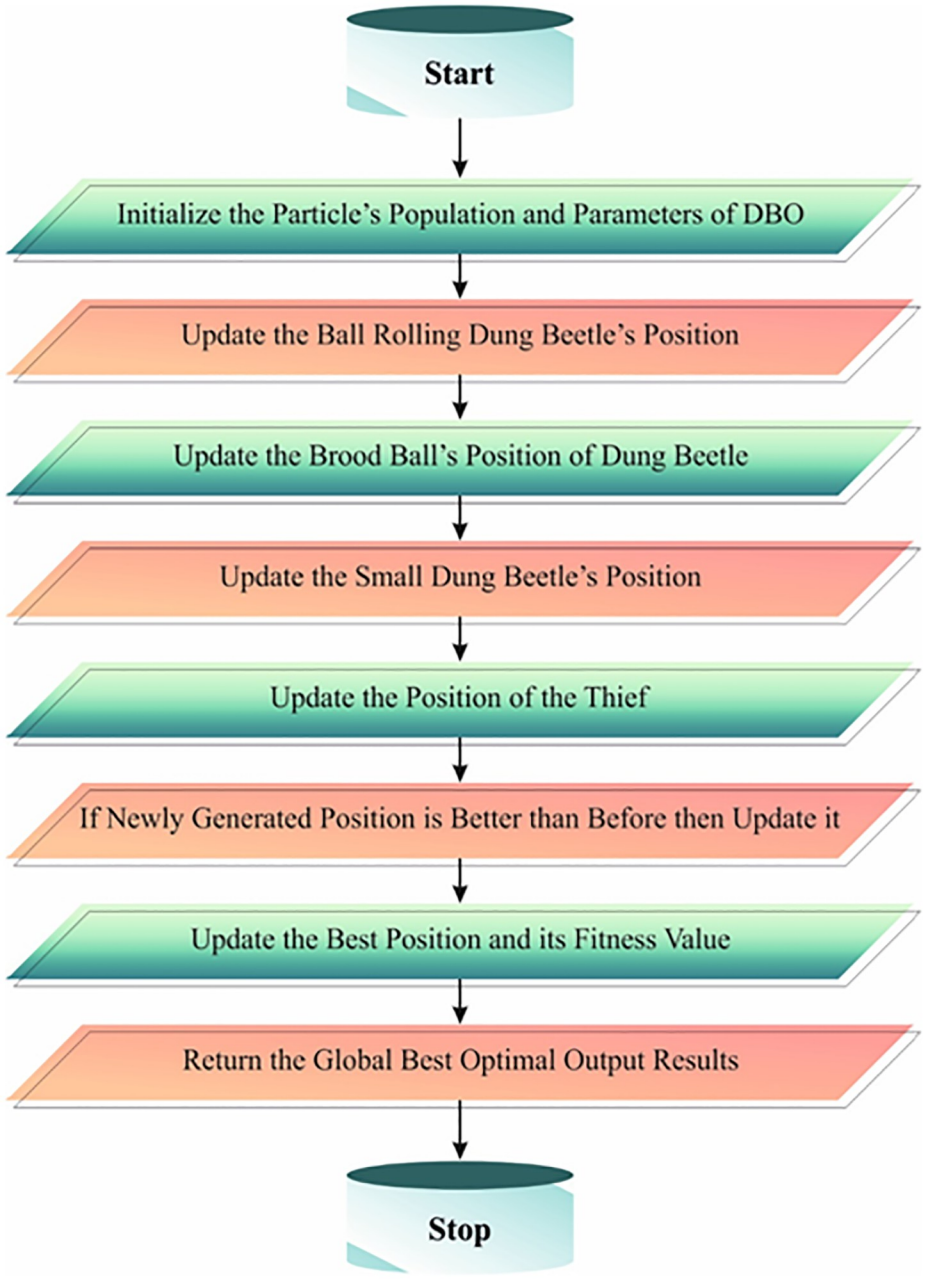
Flowchart of DBO.

Now *S* represents constant value, *x*_*i*_ (t) refers to the position data *i*^th^ stealing DB with *t*^th^ iteration, and *g* refers to a random number of dimensions 1 × *D* following a uniform distribution. Fig 2 illustrates the flowchart of DBO.

### 3.3. Process involved in scheduling process

The EASA-MORU method balances the load properly and distributes the resources based on the demands of the cloud infrastructure. A brief overview of the proposed architecture follows [37]. Consider *n* tasks as *t*_*n*_ = {*t*_1_, *t*_2_,….*t*_*n*_}, *k* VMs as *v*_*k*_ = {*v*_1_, *v*_2_, *v*_3_………*v*_*k*_}, *j* hosts as *h*_*j*_ = {*h*_1_, *h*_2_,….*h*_*j*_}, *i* datacenters, as *d*_*i*_ = {*d*_1_, *d*_2_, *d*_3_….*d*_*i*_}. Here, the model defines the problem as *n* tasks mapped carefully onto *k VMs* residing in *i* datacenter and *j* hosts while reducing the SLA violation, makespan, and energy depletion.

The cloud agent will receive and send the request to the task scheduler. Based on SLA, the task manager confirms whether the user’s request is valid. The task scheduler feeds the request to the scheduler after verifying the user request. Based on the processing capacity and length of the task, task priorities are initially calculated after the user request submission from the cloud user is elevated to the task manager level. Next, the *VM* priorities are evaluated according to the electricity price at the datacentre location. After taking priority, rankings are given for the task and fed into the scheduler for effectively assigning tasks on *VMs*. Thus, makespan, SLA violations, and energy consumption should be reduced to map tasks onto *VMs* appropriately.

Initially, the *VM* loads are computed to calculate the task priority.


$$l^{vm}=\sum \;l^{k}$$ (9)

In Eq (9), *l*^*vm*^ is the current load of *k* VMs.

Using Eq (10), the load is evaluated on the host after computing the *VM* load.


$$l^{h}=l^{vm}/\sum \;h_{j}$$ (10)

In Eq (10), *l*^*h*^ is the total load on the physical host.

Before describing task priorities, the physical host and VM loads must be evaluated. The VMs’ processing capacities *must als*o be verified, as it is vital to map appropriate tasks to suitable *VMs*.


$$pr^{vm}=pr^{no}\times pr^{mips}$$ (11)

In Eq (11), *pr*^*vm*^ specifies the processing capacities of *the VM*, *pr*^*no*^ shows the number of processing components, and *pr*^*mips*^ denotes the computation performance of the *VM*.

By using Eq (12), the processing capacity of the VM is calculated.


$$tot_{pr}^{vm}=\sum \;pr^{vm}$$ (12)

After evaluating the processing capacity of the VM, the task size must be assessed based on the following expression.


$$t_{n}^{size}=t^{mips}\times t^{p}$$ (13)

Using Eq (14), the task priority is evaluated as follows:

$$t^{pr}=t_{n}^{size}/pr^{vm}$$ (14)

The model calculates the task priority and identifies the *VM* priority based on the electricity price at the datacentre location. The high unit electricity price of the datacentre provides less priority to allocating tasks to highly prioritized *VMs*, which have the lowest electricity cost, minimizing SLA violations, makespan, and energy consumption.


$$vm^{pr}=\frac{high^{unit\;elect\;cost}}{d_{i}^{unit\;elect\;cost}}$$ (15)

In Eq (15), *high*^*unit elect cost*^ is the high electricity unit cost in all data‐centers and $d_{i}^{unit\;elect\;cost}$ is the electricity unit cost at a specific datacenter. After calculating the priorities of VMs and tasks, the study aims to minimize SLA violation, makespan, and energy depletion.

Makespan is the implementation time of the task while running on a *VM*.


$$ms^{n}=avail^{k}+e^{n}$$ (16)

In Eq (16), *ms*^*n*^ specifies the makespan of *n* tasks, *avail*^*k*^ represents the *kVMs* tasks availability, *and e*^*n*^ shows the implementation time of *n* and.

Energy consumption is the next important parameter from the cloud user and provider perspectives. Energy consumption in the cloud paradigm includes two major parts: the energy consumed during computation and the energy consumed while idling.


$$e_{con}\left(vm^{k}\right)=\int_{0}^{k}e_{con}^{com}\left(vm^{k}t\right)+e_{con}^{idle}\left(vm^{k}t\right)dt$$ (17)

Using Eq (18), the total energy consumption of VMs can be evaluated after computing the energy consumption of *VMs*.


$$e_{con}=\sum \;e_{con}\left(vm^{k}\right)$$ (18)

Next, the SLA violations must be assessed for the cloud provider and consumer because even though the SLA is violated at a specific time without completing the task by its given deadline, it results in degraded performance. First, the active time of performance degradation and physical hosts must be evaluated to evaluate SLA violations.


$$ACT=\frac{1}{r}\sum_{m=1}^{r}\frac{violation\;time_{h_{j}}}{ACT_{h_{j}}}$$ (19)

$$Per_{dg}=\frac{1}{k}\sum_{b=1}^{k}\frac{Per_{dg}^{r}}{tot_{r}^{vm}}$$ (20)

Then, the active time of performance degradation and physical host are calculated using the above equation.


$$sla_{violation}=ACT*Per_{dg}$$ (21)

In Eq (21), the metrics are defined and evaluated by using Eqs (16), (18) & (21). Using the DBO model, a fitness function is defined to enhance the parameter. Based on Eq (22), the Fitness function can be calculated.


$$f\left(x\right)=\text{min}\;\sum m\;s^{n}\left(x\right),e_{con}\left(x\right),sla_{violation}\left(x\right)$$ (22)

## 4. Experimental validation

This section [11] gives the performance of the EASA-MORU method under various measures. The suggested technique is simulated by employing the Python 3.6.5 tool on a PC with an i5-8600k, 250GB SSD, GeForce 1050Ti 4GB, 16GB RAM, and 1TB HDD. The parameter settings are as follows: learning rate: 0.01, activation: ReLU, epoch count: 50, dropout: 0.5, and batch size: 5.

**Fig 3:**
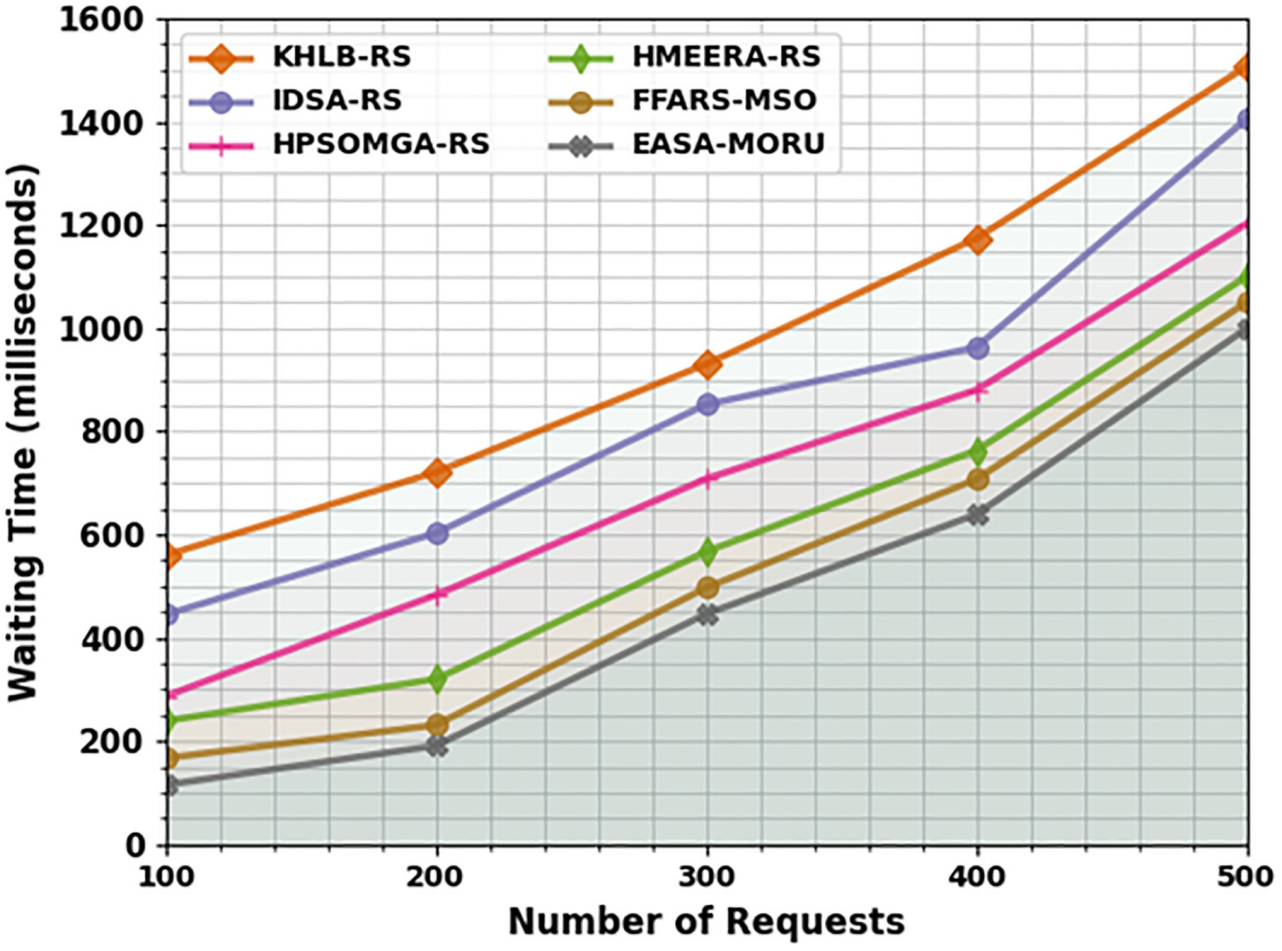
WAITT analysis of EASA-MORU technique under various requests.

In Table 2 and Fig 3, the comparative outcomes of the EASA-MORU method in terms of waiting time (WAITT) are given. The results indicate that the KH-LBRS and IDS-ARS models obtain poor performance with higher WAITT values. Besides that, the HPSOM-GARS, HMEE-RARS, and FFA-RSMSO models have reported moderately reduced WAITT values. Nevertheless, the EASA-MORU technique performs better with the least WAITT values of 115ms, 191ms, 446ms, 639ms, and 999ms under 100–500 requests correspondingly.

**Table 2:** WAITT analysis of the EASA-MORU technique with existing models on various requests.

WAITT (milliseconds)						
No. of Requests	KH-LBRS	IDS-ARS	HPSOM-GARS	HMEE-RARS	FFA-RSMSO	EASA-MORU
100	560	445	287	239	167	115
200	721	603	483	320	231	191
300	930	852	708	567	497	446
400	1174	963	880	763	707	639
500	1507	1405	1202	1100	1050	999

**Fig 4:**
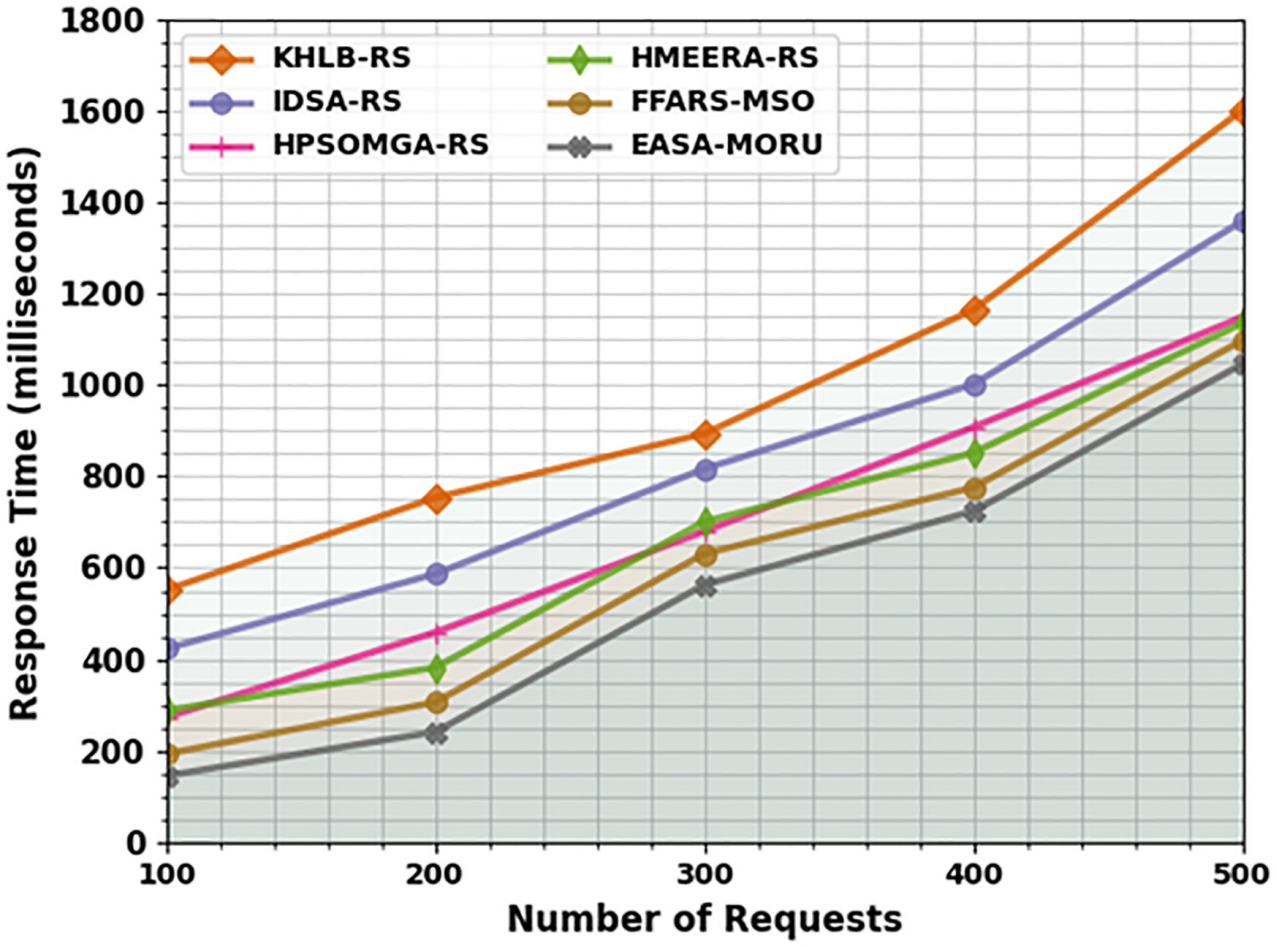
RESPT analysis of EASA-MORU technique under various requests.

Table 3 and Fig 4 show the comparative outcomes of the EASA-MORU technique in terms of response time (RESPT). The outcomes indicate that the KH-LBRS and IDS-ARS techniques perform poorly with higher RESPT values. Besides that, the HPSOM-GARS, HMEE-RARS, and FFA-RSMSO approaches have moderately reported decreased RESPT values. Nonetheless, the EASA-MORU method has superior performance with minimum RESPT values of 145ms, 241ms, 563ms, 724ms, and 1046ms under 100–500 requests.

**Table 3:** RESPT analysis of the EASA-MORU technique with recent models under various requests.

RESPT (milliseconds)						
No. of Requests	KH-LBRS	IDS-ARS	HPSOM-GARS	HMEE-RARS	FFA-RSMSO	EASA-MORU
100	552	423	273	288	193	145
200	754	587	459	382	306	241
300	894	817	680	701	631	563
400	1165	1002	908	852	776	724
500	1602	1359	1149	1136	1098	1046

**Fig 5:**
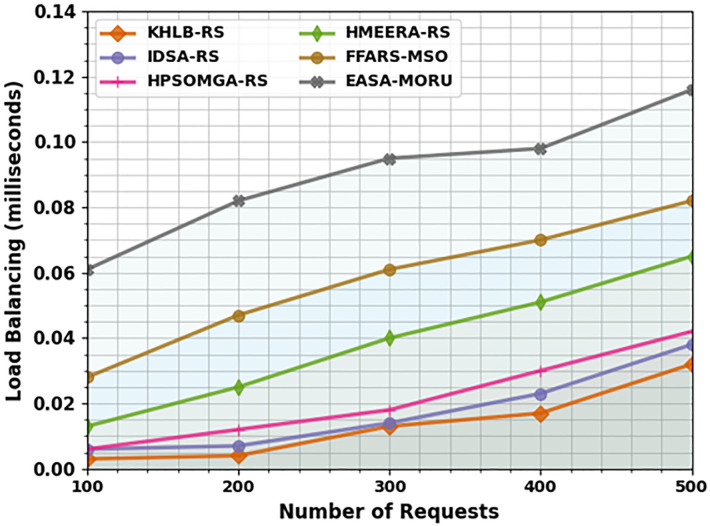
LB analysis of EASA-MORU technique under various requests.

The LB results of the EASA-MORU technique are compared with recent models under varying requests in Table 4 and Fig 5. The outcome implies that the EASA-MORU method reaches effectual outcomes with improved LB values. Under 100 requests, the EASA-MORU method gains an increased LB of 0.061ms, whereas the KH-LBRS, IDS-ARS, HPSOM-GARS, HMEE-RARS, and FFA-RSMSO models offer decreased LB of 0.003ms, 0.006ms, 0.006ms, 0.013ms, and 0.028ms, correspondingly. Also, under 500 requests, the EASA-MORU method obtains an increased LB of 0.116ms, whereas the KH-LBRS, IDS-ARS, HPSOM-GARS, HMEE-RARS, and FFA-RSMSO methods offer decreased LB of 0.032ms, 0.038ms, 0.042ms, 0.065ms, and 0.082ms, correspondingly.

**Table 4:** LB analysis of the EASA-MORU technique with recent models under various requests.

LB (milliseconds)						
No. of Requests	KH-LBRS	IDS-ARS	HPSOM-GARS	HMEE-RARS	FFA-RSMSO	EASA-MORU
100	0.003	0.006	0.006	0.013	0.028	0.061
200	0.004	0.007	0.012	0.025	0.047	0.082
300	0.013	0.014	0.018	0.040	0.061	0.095
400	0.017	0.023	0.030	0.051	0.070	0.098
500	0.032	0.038	0.042	0.065	0.082	0.116

**Fig 6:**
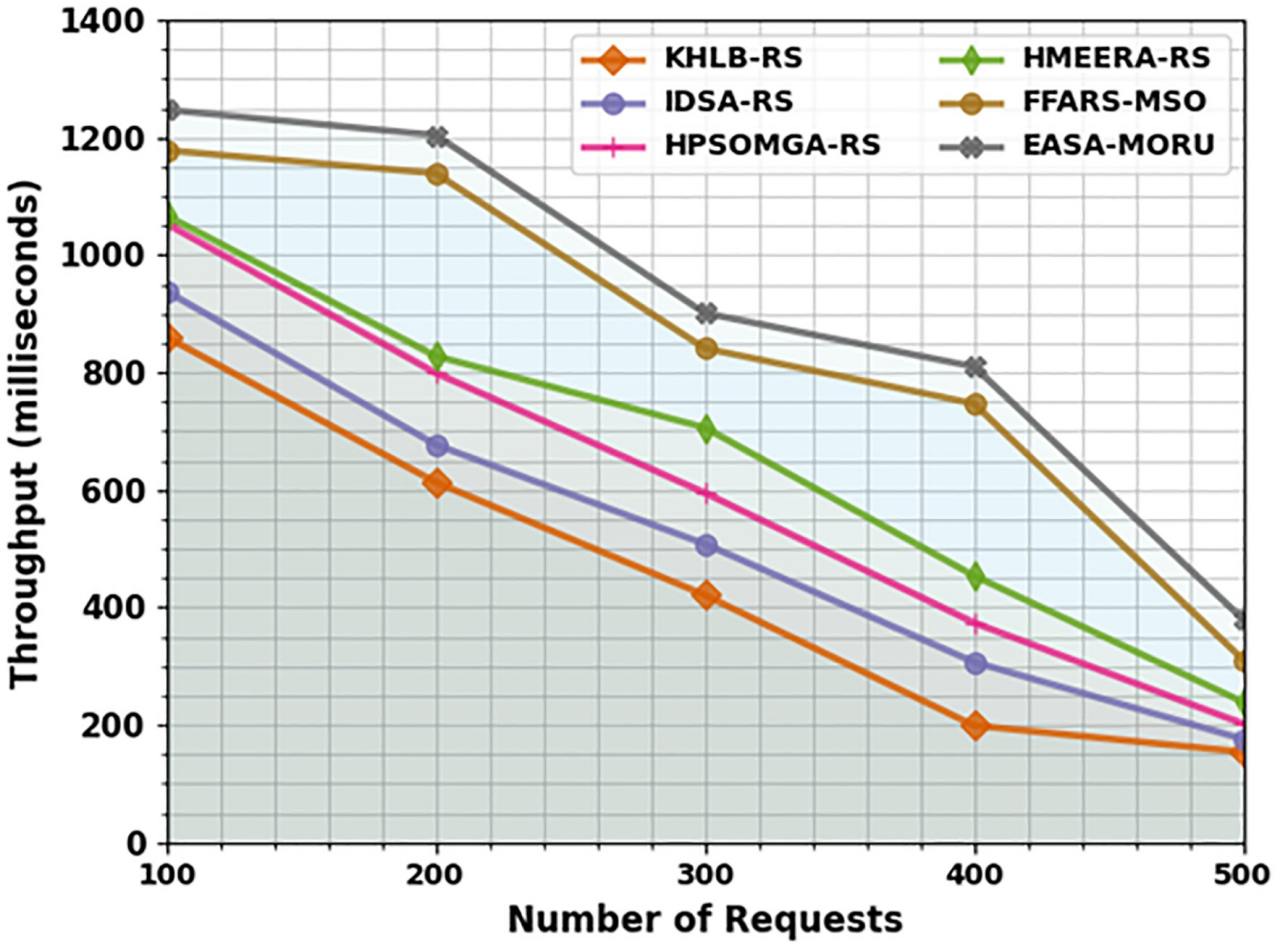
THRPT analysis of the EASA-MORU technique under various requests.

The throughput (THRPT) outcomes of the EASA-MORU method are compared with recent models under varying requests in Table 5 and Fig 6. The outcomes indicate that the EASA-MORU method reaches effectual outcomes with improved THRPT values. On 100 requests, the EASA-MORU method obtains an increased THRPT of 1247ms, whereas the KH-LBRS, IDS-ARS, HPSOM-GARS, HMEE-RARS, and FFA-RSMSO methods offer decreased THRPT of 860ms, 938ms, 1053ms, 1067ms, and 1178ms, correspondingly. Also, on 500 requests, the EASA-MORU technique gains increased THRPT of 380ms while the KH-LBRS, IDS-ARS, HPSOM-GARS, HMEE-RARS, and FFA-RSMSO methods offer decreased THRPT of 154ms, 176ms, 202ms, 239ms, and 311ms, correspondingly.

**Table 5:** THRPT analysis of the EASA-MORU technique with recent models under various requests.

THRPT (milliseconds)						
No. of Requests	KH-LBRS	IDS-ARS	HPSOM-GARS	HMEE-RARS	FFA-RSMSO	EASA-MORU
100	860	938	1053	1067	1178	1247
200	613	677	799	828	1139	1204
300	421	508	595	706	841	901
400	199	307	374	454	747	810
500	154	176	202	239	311	380

**Fig 7:**
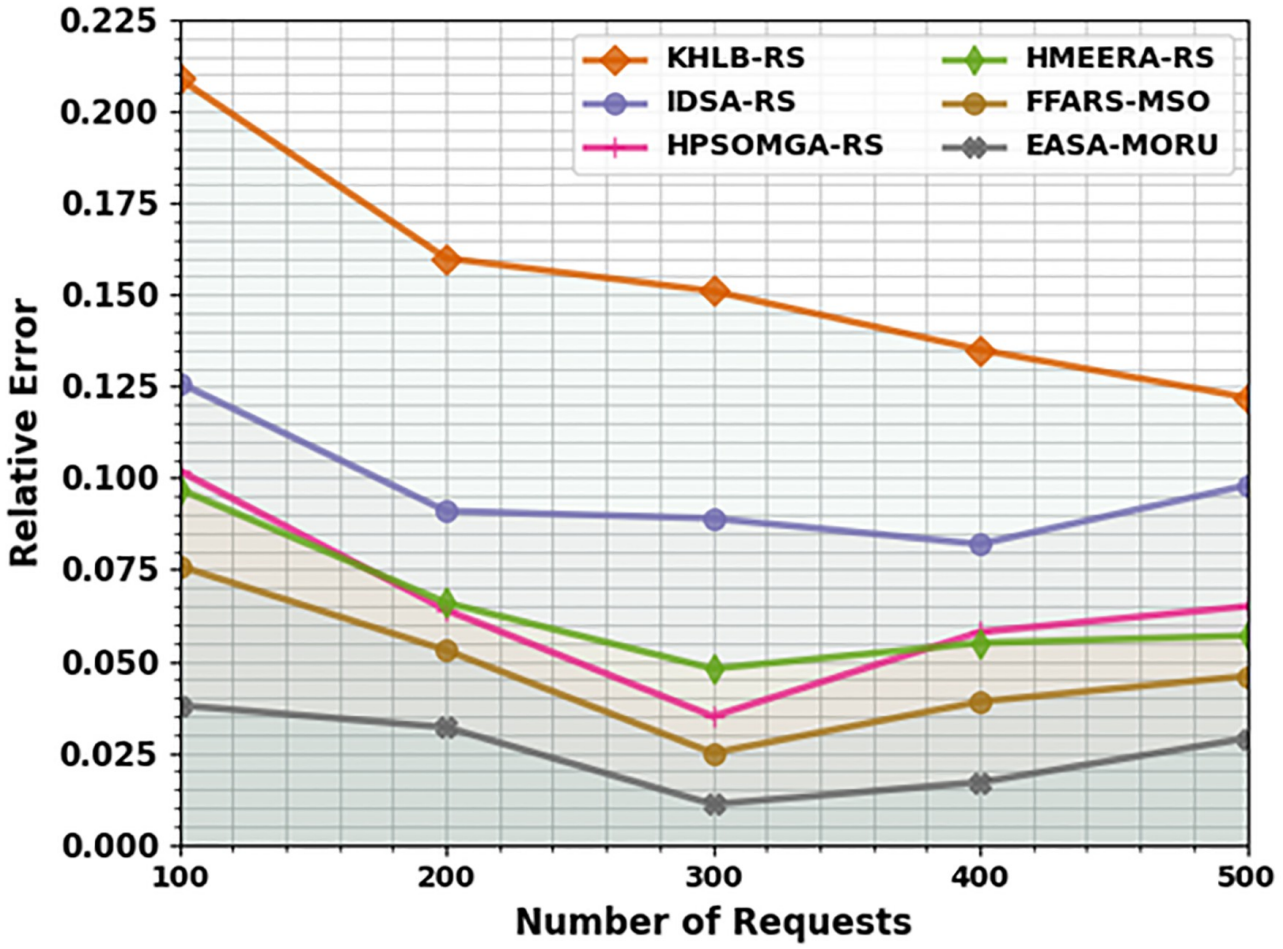
RELE analysis of EASA-MORU technique under various requests.

Table 6 and Fig 7 show the comparative outcomes of the EASA-MORU method in terms of relative error (RELE). The results indicate that the KH-LBRS and IDS-ARS models obtain poor performance with higher RELE values. Besides that, the HPSOM-GARS, HMEE-RARS, and FFA-RSMSO models have reported moderately reduced RELE values. Nevertheless, the EASA-MORU technique performs better with minimum RELE values of 0.038, 0.032, 0.011, 0.017, and 0.029 under 100–500 requests.

**Table 6:** RELE analysis of the EASA-MORU technique with existing models on various requests.

RELE						
No. of Requests	KH-LBRS	IDS-ARS	HPSOM-GARS	HMEE-RARS	FFA-RSMSO	EASA-MORU
100	0.209	0.126	0.102	0.097	0.076	0.038
200	0.160	0.091	0.064	0.066	0.053	0.032
300	0.151	0.089	0.035	0.048	0.025	0.011
400	0.135	0.082	0.058	0.055	0.039	0.017
500	0.122	0.098	0.065	0.057	0.046	0.029

**Fig 8:**
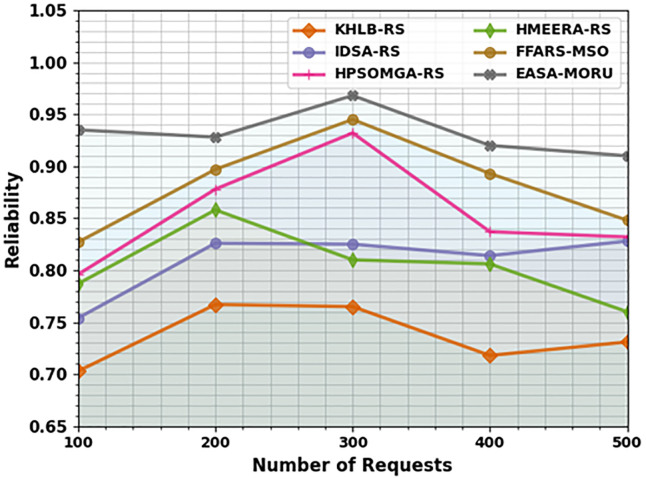
RELY analysis of EASA-MORU technique under various requests.

The reliability (RELY) results of the EASA-MORU method are compared with recent models under varying requests in Table 7 and Fig 8. The outcomes imply that the EASA-MORU technique attains effective outcomes with the highest RELY values. On 100 requests, the EASA-MORU technique attains an improved RELY of 0.935, but the KH-LBRS, IDS-ARS, HPSOM-GARS, HMEE-RARS, and FFA-RSMSO methods offer decreased RELY of 0.703, 0.754, 0.796, 0.787, and 0.827, correspondingly. Also, on 500 requests, the EASA-MORU technique obtains an increased RELY of 0.910, while the KH-LBRS, IDS-ARS, HPSOM-GARS, HMEE-RARS, and FFA-RSMSO methods offer decreased RELY of 0.731, 0.828, 0.832, 0.760, and 0.848, correspondingly.

**Table 7:** RELY analysis of the EASA-MORU technique with recent models under various requests.

RELY						
No. of Requests	KH-LBRS	IDS-ARS	HPSOM-GARS	HMEE-RARS	FFA-RSMSO	EASA-MORU
100	0.703	0.754	0.796	0.787	0.827	0.935
200	0.767	0.826	0.878	0.858	0.897	0.928
300	0.765	0.825	0.932	0.810	0.945	0.968
400	0.718	0.814	0.837	0.806	0.893	0.920
500	0.731	0.828	0.832	0.760	0.848	0.910

**Fig 9:**
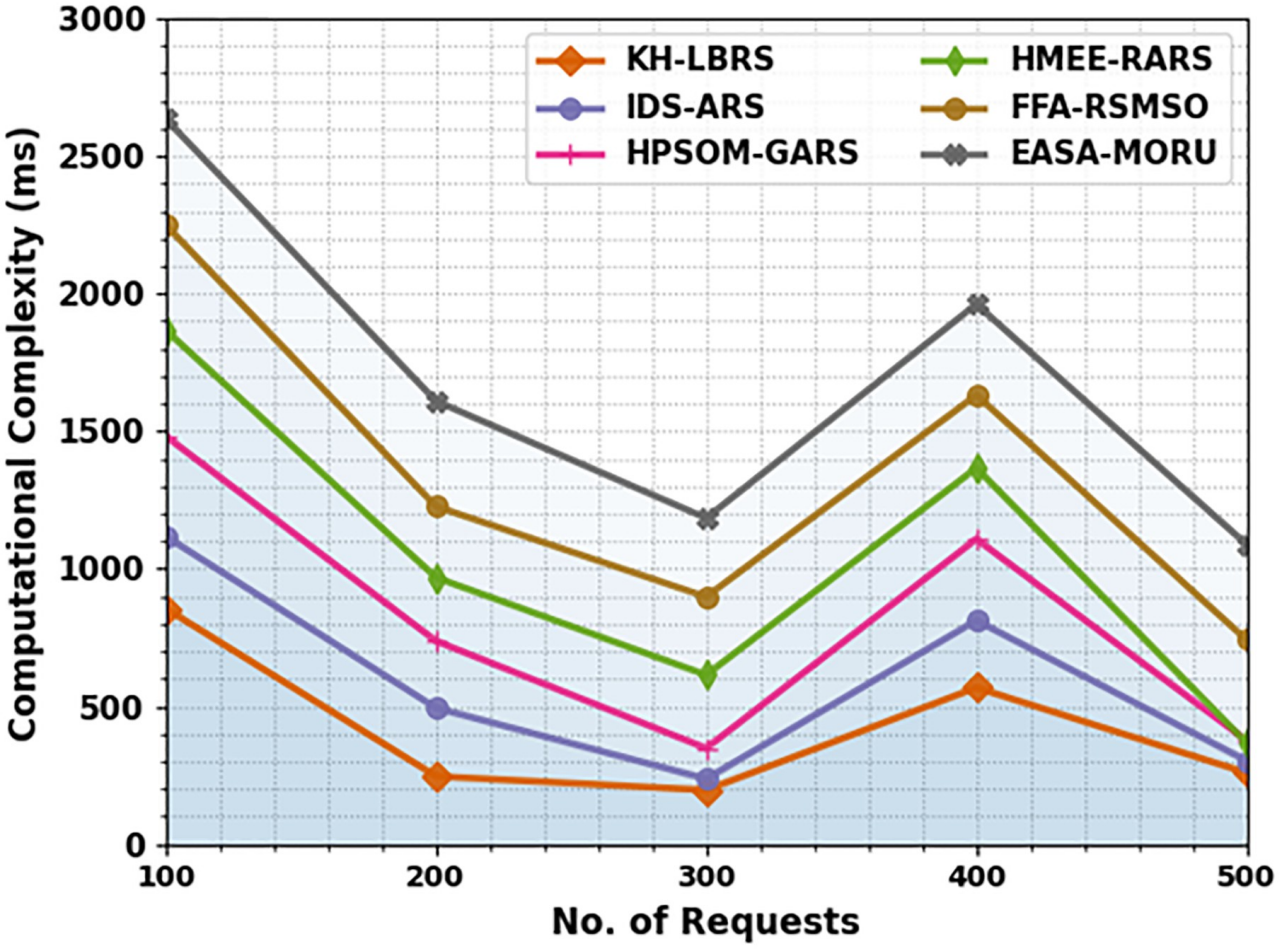
CT evaluation of the EASA-MORU model with recent technique under several requests.

The computational time (CT) outcomes of the EASA-MORU method are compared with recent techniques in Table 8 and Fig 9. The computational complexity of the scheduling models changes substantially as the number of requests increases, as depicted in the table. For 100 requests, KH-LBRS is the most efficient, with a processing time of 856 ms, while EASA-MORU is the slowest at 2634 ms. As the number of requests increases to 500, KH-LBRS maintains its relative effectualness with 260 ms, whereas EASA-MORU remains the slowest at 1090 ms. However, there is an enhancement from the initial 2634 ms. This trend illustrates that KH-LBRS consistently performs better across diverse request sizes, illustrating greater scalability related to other models. On the contrary, despite enhancements, techniques such as EASA-MORU still lag in performance, showing potential areas for optimization for handling greater datasets more effectively.

**Table 8:** CT evaluation of the EASA-MORU model with recent technique under several requests.

CT (ms)						
No. of Requests	KH-LBRS	IDS-ARS	HPSOM-GARS	HMEE-RARS	FFA-RSMSO	EASA-MORU
100	856	1118	1482	1867	2249	2634
200	247	495	738	969	1227	1610
300	197	238	349	615	899	1185
400	569	813	1109	1367	1632	1962
500	260	304	377	369	743	1090

Therefore, the EASA-MORU technique can effectively schedule the resources in the cloud infrastructure.

## 5. Conclusion

In this study, an EASA-MORU technique on cloud infrastructure is introduced. The EASA-MORU technique aims to optimize the makespan and effectively use the resources in the cloud infrastructure. The DBO method is used for scheduling purposes in the EASA-MORU technique. Moreover, the EASA-MORU method balances the load properly and distributes the resources based on the demands of the cloud infrastructure. The performance evaluation of the EASA-MORU method is tested using a series of performance measures. A wide range of comprehensive comparison studies emphasized that the EASA-MORU method achieves improved performance over other methods under diverse evaluation measures. The limitations of the MORU technique comprise potential threats in scaling to large-scale cloud environments due to the model’s sensitivity to parameter settings and convergence speed. Furthermore, exploring fusion optimization models or integrating ML methods could enhance scalability and accomplishment. Future works may concentrate on improving the technique’s robustness to dynamic workload and resource availability alterations and more effectually incorporating adaptive strategies in handling heterogeneous cloud environments. Additionally, hybrid metaheuristic models can be derived to improve the RS performance further. Besides, deep reinforcement learning techniques can be applied to extend the performance of the proposed model. Additionally, future research may include scalability analysis of the presented technique by examining it in greater, more practical instances. The model should also be investigated across diverse real-world scenarios, encompassing more tasks and resources. The adaptability and efficiency of the technique could also be shown when handling large-scale issues, which will give a clearer understanding of how well the presented technique accomplishes under the demands of real-world applications, safeguarding its robustness and efficiency in practical environments.
